# Reconceptualising knowledge in the athlete–coach learning system: a mixed-method case study of harnessing bi-directional self-organising tendencies with a national wheelchair rugby league team

**DOI:** 10.3389/fspor.2023.1196985

**Published:** 2023-10-25

**Authors:** Martyn Rothwell, Ben William Strafford, Scott Cragg, João Ribeiro, Keith Davids

**Affiliations:** ^1^ Sport and Human Performance Research Centre, Sheffield Hallam University, Sheffield, United Kingdom; ^2^ Centre for Research, Education, Innovation, and Intervention in Sport (CIFI2D), Faculty of Sport of the University of Porto (FADEUP), Porto, Portugal

**Keywords:** ecological dynamics, complex adaptive systems, knowledge transfer, team synergies, bi-directional self-organisation tendencies, constraints, principles of play

## Abstract

Knowledge and knowledge transfer are often viewed in unitary and hierarchical terms, where a linear transaction exists between an individual possessing a body of knowledge and a person needing that knowledge. Although this traditional view of knowledge transfer is common within the sports domain, it is problematic because knowledge is treated as a self-contained entity. The overarching purpose of this study is to explore the ecological role of knowledge, underpinning performance preparation processes in an international coaching setting. Specifically, we investigated how bi-directional self-organising (coordination) tendencies (coach and athlete-led) can be exploited to facilitate the formation of attacking synergies within the team sport of wheelchair rugby league. A mixed-method case study approach was employed to collect data, involving semi-structured interviews, reflexive observations and field notes, and notational analysis. Results from the study described the transitional process of positioning an ecological view of knowledge transfer as a guiding principle to enhance athlete and practitioner collaboration. This reciprocal relationship provided documented opportunities to enhance on- and off-field team synergies. The pedagogical experiences we describe emerged throughout periods of uncertainty, requiring effortful interactions, forged on the continuous coupling of key agents (individuals), content, and context, enabling application, refinement, and opportunities for team synergies to evolve in performance preparation. Results suggested that the challenge of understanding and facilitating knowledge transfer could be embedded within the ecology of a complex adaptive system, sustained as a contextualised activity reciprocally constructed through on-going correspondence between athletes, scientists, practitioners, and the competitive performance context.

## Introduction

Game-play strategy, practice designs for performance preparation, and feedback provision in team sports are essential processes that have been traditionally coach- or teacher-led. Operating in a hierarchical *top–down* approach to explicitly instruct athletes and direct tactical strategies, in what has been termed deliberate practice (1–3). Top*–*down tendencies are still pedagogically prevalent across sports, even though these approaches have attracted criticism because of the mechanistic foundations of performance suppressing the autonomy of athletes (4, 5). These insights on traditional pedagogical trends are not limited to academic researchers. A notable practical insight aligning with this strong pedagogical tendency was revealed by a New Zealand All Black rugby union player (6), when reflecting on his experiences of performance preparation at the Racing 92 club:

One of the biggest surprises I had when joining Racing was that everyone did what the coach said. In team meetings, players would not say a word … I had to bite my tongue. There was no awareness of playing what you see. My career has been about backing my instinct and being prepared to go against the gameplan.

Not all pedagogical approaches are predicated on traditional, explicit, coach-led game-play strategies, and a large body of literature advocates the (re)conceptualisation of team sports as a complex adaptive system (CAS) (7). In a CAS, athletes, coaches, support staff, and the environment are mutually entwined and reciprocally influence performance behaviours towards achieving a specific goal (8, 9). This (re)conceptualisation of performance in team sports is founded on a complex systems view of human behaviour, describing how collective team play and tactical formations emerge under specific constraints (10, 11). During a team sport competition, surrounding constraints (e.g., tactical principles of play) can facilitate the co-adaptation of players to form rich patterns of behaviour that configure synergy formation (e.g., combining actions) between players (12). Whilst satisfying a range of constraints, individuals differentiate between sources of information that can specify relevant affordances (their opportunities for action), available to be utilised during a competitive performance (13). Individuals and teams with the functionality to select relevant affordances whilst satisfying constraints are likely to have the adaptability to solve performance problems and regulate their actions supported by the guidance of an external source (e.g., a coach). However, despite a growing body of research conceptualising teams as CAS, case studies documenting how practitioners have used this concept as a framework to inform their practice are lacking (14).

Without these important applied insights on contemporary approaches to practice, longstanding issues will remain. For example, a challenge for coaching practitioners is understanding how to provide conditions in practice and competition that support athletes in satisfying global constraints (e.g., prepared game-play strategies) and local constraints (e.g., co-adaption between teammates as contexts change) for enhancing synergy formation. Although there has been a traditional tendency to polarise these two dichotomous positions within an applied practice, Ribeiro et al. (15) provided a substantial theoretical rationale suggesting that global-to-local (e.g., top*–*down) and local-to-global (e.g., bottom*–*up) self-organisation (coordination) tendencies can *co-exist* and be intentionally integrated to enhance performance in team sports. Ecological dynamics emphasises that these bi-directional tendencies exist on a heterarchical continuum. Framed in a more flexible approach, coaches and athletes may conceive less rigid global influences as *principles* to guide intentions and actions of players in team invasion sports (e.g., advance forward, attack space between and behind defenders, and support the ball carrier can form the basis of attacking play). Using coach and athlete interactions to co-design practice tasks can facilitate the formation of stronger, adaptive, and less predictable synergies between players (16). More fluid global influences of this nature support players to co-adapt their actions under localised sources of information (e.g., immediate teammates and opposition actions), therefore becoming less reliant on global and explicitly pre-determined ideas. From a CAS perspective, the process of synergy formation in the athlete–coach–environment system means that spontaneous changes in competition increase the probabilities for adaptive behaviours to evolve (17). With this tendency in mind, training programmes must be flexible, contain uncertainty, and offer opportunities for local-to-global self-organising tendencies between players to emerge and strengthen athlete–coach–environment interactions (18).

Regardless of these advances in understanding bi-directional tendencies in team sports performance, there are few examples of how coaching practitioners have implemented these theoretical insights in practice. A reason for this paucity could be the challenges associated with transferring sports science research, theory, and data into applied practice. For example, Buchheit (19) argued that some sports scientists may lack sport-specific knowledge to appreciate the performance problems that need solving. Indeed, this dilemma has stimulated a wealth of academic publications that have aimed to unpack the nuances associated with applying research into practice [e.g. (20)]. However, all too often, scholarly activity aimed at understanding this challenge creates a false dichotomous view of the disconnect between the academic world (e.g., research to satisfy universities’ strategic objectives that may have little applied value to practitioners) and research that can support practitioners to solve immediate performance problems (19). Whilst this point has been well made, rather than viewing theory and practice in binary terms, time may be better spent exploring how the closely connected and intertwined nature of theory and practice can facilitate the reciprocal exchange of empirical and experiential knowledge, which can support the generation of performance solutions in competitive sports contexts. As Ross et al. [(21), p. 2] have commented, bridging the research–application divide is an immediate challenge that needs attention from both sports scientists and coaching practitioners to provide an “integrative blend of evidence-based practice and practice-based evidence”. Better research–application integration can aid sport performance practitioners in answering fundamental questions that provide insights into what conditions may facilitate creative and functional behaviours for high performance.

To that end, this paper aims to explore the challenge of knowledge transfer in a high-performance coaching setting. More specifically, we sought to address the calls of Ribeiro et al. (15) to explore how the application and interplay of bi-directional tendencies can be harnessed for the self-organisation of emergent attacking behaviours in a team sport. Academic contributions have provided detailed theoretical insights into bi-directional tendencies. However, to date, no empirical studies have positioned a bi-directional synergy formation approach to training and performance preparation. Whilst current contributions to the CAS literature provide the academic community with theoretical insights, little effort has been made to support sport scientists and practitioners’ understanding of how local-to-global tendencies can be harnessed to develop adaptive team performance, diversifying tactical patterns of behaviour as competitive performance conditions change (15). Difficulties associated with implementing studies of this nature in real training and competitive contexts (e.g., time constraints, squad availability and access, and coaches’ authorisation) have limited the understanding of contemporary applications. Therefore, this study offers a novel contribution, hoping to motivate sports scientists and practitioners to implement future studies that explore novel approaches to performance preparation based on bi-directional synergy formation. To achieve the overarching purpose, a mixed-method case study approach was deemed appropriate to address the following aims: (1) document the experiences of the first author in creating conditions that facilitate a balance between bi-directional self-organising tendencies in a high-performance sports setting; (2) explore how the reciprocal interactions between players, coaches, and contexts shape the emergence of team synergies; and (3) collect and analyse performance data to provide an additional opportunity to facilitate the exchange of reciprocal knowledge between players and coaches. Typically, performance analysis methods remove the athlete from the process, rendering them redundant and mere recipients of information to critique their performance. More specifically, aim (3) used descriptive analysis to encourage stronger interactions between the wider coaching team and the players to discuss and evolve how implementing bi-directional tendencies can facilitate attacking strategies.

## Materials and methods

### Research design

Although there are different ways to face the challenge of understanding the knowledge transfer process in sports, this study chose the mixed-method approach to provide flexibility in using quantitative and qualitative methods to address the common research goal (22). In addition, a case study design was selected because, as Simons [(23), p. 21] suggested, a “case study is an in-depth exploration from multiple perspectives of the complexity and uniqueness of a particular project, policy, institution, program or system in a ‘real life’ context.” More specifically, in the same way that Hodge et al. (24) examined the motivational climate created by the New Zealand Rugby Union team, an instrumental case study was undertaken to achieve that purpose. The Sheffield Hallam University ethics committee provided ethical approval. The data collection period spanned two and a half years. It involved collecting data from casual conversations, semi-structured interviews with the athletes, first author reflexive observations and field notes, and analysis of performance statistics collected from international fixtures.

### Positionality and reflexivity

At the outset, it is important to acknowledge that the first author’s (in)experience as a coach and academic will play a significant role in the production of this research. Whilst many years of working in coaching, coach education, and player development provided MR with a physical–cultural insider status, providing many advantages in securing access to the field (25), it also presents considerable challenges regarding researcher subjectivity. In addition, MR’s practice (together with the co-authors’ theoretical persuasion) was informed by an ecological rationale of sports performance, positioning athletes and teams as complex adaptive systems who self-organise their collective performance behaviours (9) under interacting task, organismic, and environmental constraints. We acknowledge that because of the history and theoretical positioning of MR, interpreting experience (and data) can never be free of value. However, as Blackshaw (26) argued, these biographical experiences enable the interpretation of the phenomena under study. To challenge these subjective assumptions and accept that the research team cannot be detached from the research process, all stages adopted reflexive practice. This approach included facilitating discussions with players, coaching and professional support staff (other insiders), and academic colleagues who were outsiders to wheelchair rugby league, intending to maintain a critical and reflective perspective. Standing back from the analytical process and being reflexive and self-critical supported the research team in providing methodological rigour, challenging their theoretical assumptions and analytical conclusions (26). Adopting reflexive practice provided opportunities to question theory, reflect on our own assumptions of practice design and performance preparation, and be self-critical towards our interpretations of the qualitative findings [e.g. (27)].

### Context and background

The first author (MR) was situated as an assistant coach with a national wheelchair rugby league team, a sport played by mixed physical ability and mixed gender athletes. MR joined the coaching team in January 2020 and was tasked with preparing the playing squad for the 2021 World Cup competition (played in 2022 because of COVID-19 restrictions). The old coaching team had been overseeing the development of the playing squad for the previous World Cup cycle (4 years), and all previous performance staff were no longer part of the new coaching group. Initial observations of typical practice and competition performance tendencies (influenced by the old coaching team) and casual conversations with players indicated that practice designs and match play tactics were dominated by a global-to-local direction. This approach to practice was characterised by a strict adherence to a structured game model where rigid, predictable, and inflexible on-field behaviours limited the co-adaptation and emergence of synergistic relations between players. For example, during the attacking phases of play, specific players advanced the ball to precisely specified positions on the field of play, pre-determined by a numbering system (e.g., the pitch was split into channels, numbered 1–5 from left to right). Once a player had advanced the ball to the “correct” pre-determined position, they became redundant during play until their next pre-planned involvement.

Initial observations of this approach suggested that it was over-orchestrated, with the propensity for global system behaviours to explicitly over-constrain player interactions and co-adaptation in a top*–*down fashion. Overvaluing the influence of global-to-local tendencies made some of the current playing squads assume that they could not be the source of their own activity. In other words, the players believed that they could not effectively interact with the opportunities for action that emerged in competition without the explicit instructions or guidance of the coaching staff, or a rigid game model [for a theoretical explanation of human behaviour in ecological psychology, see (28)]. Indeed, the existing performance model failed to exploit bi-directional self-organising tendencies, disengaging some players from the learning, development, and performance preparation process.

Several months before the case was studied, the new coaching team and players began to facilitate performance preparation for the international competition by introducing the key idea of exploiting both bi-directional tendencies in practice and competition. More specifically, the previous structured and rigid game model was refined, and attacking team play and practice designs were based on flexible principles of play (termed *Go Forward with Support*) co-created by the players and coaching team [which included guidance to work in pairs, explore, and vary play to play (early pass, change of direction, and tempo); all players “stay alive” on every play]. Coach observations and athlete insights of this approach indicated that local interactions were heightened through players being given more freedom to explore the global principles of play, according to their unique capacities and characteristics, which ultimately afforded greater synergy formation at a local level. A critical factor in this process was drawing on the rich experiential knowledge of this group of international players to identify these principles. Adopting this approach allowed the players to share their detailed insights of the game. Therefore, principles were based on the needs of the players, action capabilities, *knowledge of* the performance environment, and its affordances in competition [e.g. (13)]. Woods et al. (29) conceptualised this approach as *representative co-design*, suggesting that drawing on an athlete’s experiential knowledge is vital in strengthening local self-organising interactions between teammates and opponents, an important factor in enhancing team synergies (30).

### Participants

The participants were all part of a national wheelchair rugby league team preparing for the 2021 World Cup competition. The age of the athletes (*N* = 14) ranged from 18 to 36 years (*M* = 28.3, SD = 6.18), and international appearances ranged from 6 to 30 games (*M* = 18.9, SD = 8.25). Prior to data collection, the proposed research period was presented to the players during a team meeting. Following a series of questions about the study, all participants provided informed consent before data collection took place.

### Semi-structured interviews

Semi-structured interviews were selected as one data collection method so that MR and the players could co-create insights relating to the aim of the study (31). Individual interviews were conducted face-to-face with all 14 players and ranged from 35 to 80 min in duration (*M* = 56.8, SD = 13.4). Interviews were conducted with the players between September 2021 and September 2022, prior to the start of the World Cup competition. Some interviews were conducted during training and competition periods, with others taking place away from formal settings. Approaching the interviews this way ensured all players had time to experience the balanced approach between self-organising tendencies in practice and competition. During data collection, all interviews were audio-recorded and transcribed verbatim. The specific purpose of the interviews was to explore the participants’ experiences of practice and match play conditions that facilitated performing under local-to-global self-organising tendencies. The interview guide posed questions relating to general experiences (e.g., “Can you tell me about your experiences of playing without a structured game plan?”); more focused experiences, such as individual changes (e.g., “Has playing without a structured game plan changed how you see and act on opportunities?”); and interactions with teammates and opposition players (e.g., “Have you noticed any changes in your interactions with teammates and opposition players?”). In addition, probe questions were used to explore these areas and player responses in further detail.

### Reflexive observations and field notes

Reflexive observations and field notes were used in conjunction with the semi-structured interviews to develop a deeper understanding of facilitating a balance between bi-directional self-organising tendencies (32). Observations occurred before, during, and after training sessions and competitive matches, in team meetings, and during video review sessions. All these settings presented an opportunity to observe players, coaches, and support staff reactions, comments, attitudes, interpretations, acceptance, and resistance to practising and performing under bi-directional self-organising tendencies [e.g. (33)]. Immediately after these events, field notes were made away from the team environment (e.g., hotel room, quiet areas at the training venue, and a local café) to record observations and experiences pertinent to the aims of the study. Whilst reflexive practice was a golden thread throughout all stages of the research process, it was important here so MR could reflect on how his subjectivities and theoretical assumptions informed interpretations of observed events [e.g. (34)].

### Performance analysis

Notational analysis was conducted to examine how attacking synergy formations emanating from both bi-directional tendencies influenced collective defensive behaviours. Recordings from four international games were analysed. The attacking team play from two of the games analysed had a strong global influence (under the previous coaching team). The other two games represented attacking team play favouring more local-to-global self-organising tendencies (the team had been performing under more local influenced play for approximately 12 months prior to data collection). A total of 204 tactical events were sampled across the matches, as these fulfilled the criterion of having complete data for every event series.

### Notational analysis

The notational analysis data were recorded using an *ad hoc* observational instrument created in Nacsport. To ensure the stability of notational data, the observational instrument was developed using operational definitions (Table 1) and indicators of key performance adapted from empirical research on rugby union (35). It is important to note that an optimal value on defensive positioning cannot be achieved since the interaction between attacking and defensive situations changes from second to second. Regarding defensive stability, we operated within a bandwidth of suitable actions. For example, a player facing their own goal line or not part of the defensive line would be coded as an unstable defensive position. Video footage of each game was scrutinised using freeze frame functions and playback speed in Nacsport, allowing all tactical actions to be compared against the operational definitions (36).

**Table 1:** Operational definitions.

Defensive stability	
Great instability	Two or more players are out of position when the tackle is made or when a play the ball is completed to restart play.
Moderate instability	One player is out of position when the tackle is made or when a play the ball is completed to restart play.
Stable	No players are out of position when the tackle is made or when a play the ball is completed to restart play.
Tackled by number of defenders	
Tackled by one defender	A single defender committed to tackling the ball carrier.
Tackled by two defenders	Two defenders committed to tackling the ball carrier.
Tackled by three defenders	Three defenders committed to tackling the ball carrier.
Play the ball	
Play the ball	The play the ball consists of the tackled player facing the opponents’ goal line, placing the ball on the floor, and promoting the ball backwards. The ball is deemed in play when it moves backwards.
Play the ball speed	The time it takes for the play the ball to be completed.

### Data analysis

#### Reflexive thematic analysis

Data collected through field notes and semi-structured interviews were analysed through reflexive thematic analysis (RTA) (37). Using this approach, MR engaged in a reflexive, thoughtful, and non-linear manner to generate themes related to the study aims (38). An important point to note here is that for researchers working with RTA, the aim is not to provide “accounts of accurate or reliable coding” to generate a single truth but to spend time immersed in context and data to systematically interpret information and generate themes [(37), p. 1393]. As such, Braun and Clarke’s (39) six-stage approach (e.g., familiarisation with the data, generating initial codes, generating themes, reviewing potential themes, defining and naming themes, and producing the report) served as a framework to support an iterative process, where semantic and latent coding was used for theme generation. More specifically, and in no particular order, MR would read transcripts multiple times, make and revise notes, consult with interviewees when clarification was needed on specific themes, refer to theory, and share ideas with the co-authors to scrutinise and develop themes. Through this process, a final set of themes was generated.

#### Performance analysis data

Descriptive analyses were employed in Microsoft Excel to calculate absolute frequencies for each variable. Using descriptive statistics during the analysis aimed to provide clear and collaborative coaching practice. In contrast to using inferential statistics indicating an association or difference between two or more variables, descriptive statistics were considered more effective in displaying data to the coaches and players because of the ease of summarisation and interpretation of data (40).

## Results

### Observations, field notes, reflections, and notational analysis

This subsection presents findings that combine the first author’s observations, field notes, reflections, and performance analysis data to document the facilitation of a learning system to introduce bi-directional self-organising tendencies during practice and competition. As such, this subsection will adopt a predominately first-person perspective to reflect personal experiences of this challenge. The lived experiences of MR are presented to highlight the value of working through an athlete–coach learning system to challenge the status quo, and how generality and specificity of practice, and notational analysis data encouraged a balance between bi-directional tendencies in training and competition.

### Enhancing player and practitioner collaboration through transdisciplinary practice

When a coach is faced with the challenge of changing deeply rooted socially and culturally informed ways of performing and preparing, they have to make several tricky and important decisions. First, how does the coach start to identify and challenge factors that serve to maintain the status quo and generate organisational inertia, without disrupting team cohesion (i.e., team performance)? Second, what positionality will the coach adopt, and subsequently, what type of relationships will be developed with athletes as they navigate hierarchical team structures and rigid processes that inhibit alternative performance preparation methods? Third, what technological tools could support the process of evolving collective performance behaviours? Deciding how to address these key challenges required continual individual and group reflection, discussions with the players and wider coaching team, and a continuous process of engaging with scientific literature. The latter provided an important point of academic insight to guide self-reflection and critical thought and, therefore, better equipped MR to challenge accepted and normalised ways of performing.

Woods et al.’s (16) alignment of transdisciplinary research for sports science research and practice was adapted to (re)position my role, intentions, and interactions with the wider team. Quite quickly, based on hierarchical thinking and siloed practice, I realised that adopting a coach-led approach would not engage this group of players to embrace alternative ways of performing. I was concerned that they would become too reliant on me to solve performance problems and instruct them on what to do during competition. Deciding on how I would position myself within the wider team (athletes and the extended support staff group including the Head Coach, sport scientists, and performance analyst) was essential to change playing methods established within the sport for many years. Adopting a transdisciplinary approach (e.g., the framing of a competitive performance problem or challenge through the consideration and integration of fundamental principles), the wider team was encouraged to temporarily park their preconceived ideas about what it means to perform and collaborate to consider new opportunities (framed by collective principles to support competitive performance) aimed at surpassing current levels of performance. Adopting this view of integrating principles of performance reduced the effects on professionals working in disciplinary silos and placed a collective inquiry focused on the heart of the programme. This integrative approach was crucial to supporting a heterarchy of practice where all members of the wider team were given equal standing irrespective of playing experience or time spent as a practitioner. This contemporary approach supported the implementation of a transdisciplinary focus to integrate efforts confronting the problems and challenges that were likely to face the team in competition.

Initially, players were reluctant to contribute their ideas and challenge norms; after all, they would be challenging the very people responsible for their selection into the final World Cup squad. Therefore, although this power balance was never neutral, over time, players did start to offer their insights to evolve playing strategies. A pitfall of this approach was that the stronger (more confident) characters within the playing group tended to dominate conversations in group messaging chats and during team meetings specifically aimed at idea sharing. At certain points across the programme, these players were asked not to contribute in group settings but share ideas directly with the coaches away from shared forums. This approach also meant that these players became more confident in challenging me and other staff to highlight areas of contention. At first, I felt uncomfortable with this and assumed that I had a more informed perspective. However, as one player pointed out to me, their current experiences of the sport present a different perspective to mine, and just because his view did not align with my thinking does not signify that it has no value. This player–coach interaction highlights the dynamic nature of sport (and therefore the need for adaptability), where rules, physiological demands, tactics, and skill requirements can evolve and change from season to season. This means that a coach’s perspective of a sport they played several years ago might be very different to a player’s present-day experience.

This interaction is one example of the challenges experienced by the wider team when developing a reciprocal and collaborative relationship. Over time, this approach did open new areas of inquiry that could be explored together, contributing to the evolution of team performance (i.e., enhancing team synergy formation to coordinate efforts). To exemplify, during a team performance analysis session, a player highlighted his frustrations with playing across a balance of self-organising tendencies. He argued that team synergies during attacking phases were not as effective as playing under global influences (a structured game plan), raising concerns over losing momentum during the attacking phases of play. This point is highlighted here:

At full pace (playing under global influences), and the times we did score is when we offloaded because we had support, then we were an actual threat because we’ve got power and we’ve got momentum that we can use, and we lost a lot of momentum (playing under local influences). If you go back through a lot of the sets, we did I don’t know three out of five tackles we lost momentum because we either did a shit pass to someone who was at a stop, and he didn’t really go any further, or we settled down or we decided to keep it (play safe). (Player 10, Interview data)

This interaction is just one example of where notational analysis data were used to open up further communication channels between the wider coaching team and the playing group. The players’ qualitative insights and notational analysis data (Table 2) were used to frame a discussion that explored ways to evolve collective team attacking strategies. Based on the idea of momentum (identified by player 10), Table 2 indicates attacking tendency and play the ball speed, which is considered an important part of gaining momentum in rugby league (41).

**Table 2:** Play the ball speed for global-to-local and local-to-global tendencies.

	Tendency	
	Global-to-local	Local-to-global
PTB speed (s)		
0–2	7.35%	11.27%
	(15)	(23)
2–3	15.69%	14.71%
	(32)	(30)
3–4	13.73%	12.26%
	(28)	(25)
4–5	8.33%	3.92%
	(17)	(8)
5–6	1.96%	2.45%
	(4)	(5)
6+	5.88%	2.94%
	(12)	(6)

Total actions = 204, and data are reported as absolute frequencies and relative frequencies.

Whilst play the ball speed of 0–2 s could be considered the most effective way to perturb defensive stability to gain attacking momentum, this attacking indicator did not occur frequently (local-to-global 11.27%; global-to-local 7.35%). The most common play the ball speed was between 0 and 4 s under both self-organising tendencies. Alongside these objective insights, the qualitative insights of the playing groups were drawn upon identifying key properties related to enhancing play the ball speed. Following this interaction, the players were tasked with identifying factors that led to the team losing momentum (factors that notational analysis did not provide). Players collated these responses and presented them back to the wider group during a team meeting. As discussed next, these key properties provide practice designs aimed at enhancing team synergies.

### Enhancing team synergies within the athlete–coach learning system

Following an agreement between playing and coaching staff regarding match play and practice conditions encouraging exploration through elaborating flexible principles of play, I was faced with the challenge of designing practice conditions to facilitate the exploration of self-organisation tendencies through a heterarchy of bi-directional tendencies. Observations and reflections of practice that aimed to exploit bi-directional tendencies indicated that some players remained dependent on the certainty of having a very prescriptive way of playing. The collective team behaviour within these practices could be categorised as non-cooperative, revealing poor inter-player connectivity. I was surprised at how dependent some players had become on global influences (prescriptive instructions, verbal feedback, and detailed pre-determined performance plan), which made the challenge of integrating the two self-organising tendencies a difficult one. How could the coaching team start to break this dependency? What practice methods could be designed to encourage players to coordinate and regulate actions at a local level? An obvious starting point was aligning practice to the co-created principles of play by *guiding their intentions* (42). This approach could provide players with opportunities to use their unique capacities to explore adaptable and innovative performance solutions. However, practice that aligned with the principles of play encouraged the more dominant and confident players to act as a global influence on collective team actions. During practice, these players instructed, commanded, and directed their peers to carry out specific actions to achieve certain task goals. Although the coaching team encouraged a more flexible approach to exploring the principles of play, other influences (i.e., dominant players) meant that the balance between bi-directional tendencies was still dominated by global levels of influence. The potential for synergy formation to emerge from transactions between all the players in the squad was still inhibited. At this stage, identifying how to design practice conditions to exploit both bi-directional tendencies felt like an impossible task.

To improve the players’ capability to adapt to bi-directional tendencies, it was important to delve into the applied scientific literature on networking in team sports. Orth et al.’s (43) conceptualisation of an *athlete–coach learning system* provided a framework to form co-adaptive relationships with the players. The framework proposes that a reciprocal and co-adaptive relationship is formed with the coach, athlete, and performance context, constituting a *learning system*. In this system, the coach and athlete continually collaborate to form couplings from which information emerges to facilitate *in situ* task constraint manipulation. Through this approach, new opportunities for skilled action, learning, and functional performance can form. Orth et al.’s (43) conceptualisation guided my thinking and actions, acknowledging that the learning process and any changes to the current pedagogical methods needed to be based on a mutually reciprocal co-adaptive relationship between the players and the coaching team. In other words, the process of learning and change was mutually dependent, in that it could not emerge in isolation because any changes within the learning system were based on interactions between the players and the coaching team. Crucially, the coaching team’s use of notational analysis data and players’ qualitative insights of playing under local influences highlighted the performance benefits of exploiting bi-directional tendencies (integrating top*–*down and bottom*–*up influences).

To exemplify, Table 3 presents the summary data to indicate the number of defenders tackling the ball carrier and the levels of defensive stability at the point the tackle was made. Tackles made by one defender (global-to-local, *n* = 81; local-to-global, *n* = 48) led to more stable defensive outcomes (*n* = 101), compared with moderate instability (*n* = 45) and greater instability (*n* = 3). Tackles made by two defenders had equal distribution between both tendencies (*n* = 27), leading to more moderate instabilities (local-to-global, *n* = 16; global-to-local, *n* = 14) compared with stable outcomes (global-to-local, *n* = 13; local-to-global, *n* = 10).

**Table 3:** Attack and defensive outcome for global-to-local and local-to-global tendencies.

	Tendency					
	Global-to-local	Local-to-global	Global-to-local	Local-to-global	Global-to-local	Local-to-global
	Defensive outcome					
	Great instability		Moderate instability		Stable	
Attack outcome						
Tackled by one defender	0.49% (1)	0.98% (2)	9.80% (20)	12.25% (25)	29.41% (60)	20.10% (41)
Tackled by two defenders	0% (0)	0.49% (1)	6.86% (14)	7.84 (16)	6.37% (13)	4.90% (10)
Tackled by three defenders	0% (0)	0% (0)	0% (0)	0.49% (1)	0% (0)	0% (0)

Total actions = 204, and data are reported as absolute frequencies and relative frequencies.

The use of data helped shift players’ intentions, with greater value and meaning being placed on a more flexible way of playing (emphasising bottom–up influences a little more). Co-adaptive and tightly coupled interactions started to emerge through carefully considered constraint manipulations, based on emergent information emanating from the athlete–coach learning system (43). Here, the wider team collectively searched for, discovered, and manipulated constraints specific to the needs of individual and team behaviour.

**Figure 1:**
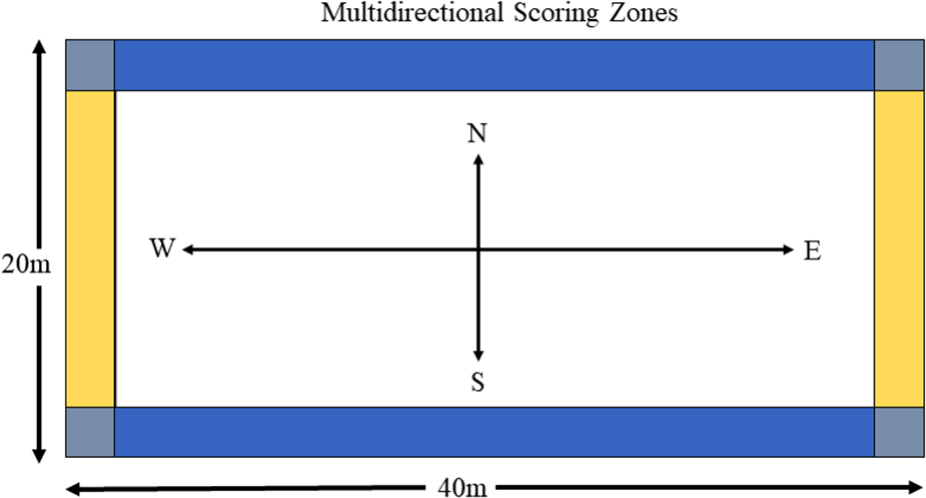
Two teams of 5 vs. 5 play against each other. Team 1’s direction of play is north to south, scoring in the blue zones. Team 2’s direction of play is east to west, scoring in yellow zones. Other than the normal game rules, no instructions are provided to the players. On the coach’s signal (long whistle blast), teams change the direction of play and therefore the scoring zones. The game requires collective team (re)organisation to facilitate fast and multi-dimensional transitions to maintain defensive and offensive advantages, encouraging self-organisation at a local level.

**Figure 2:**
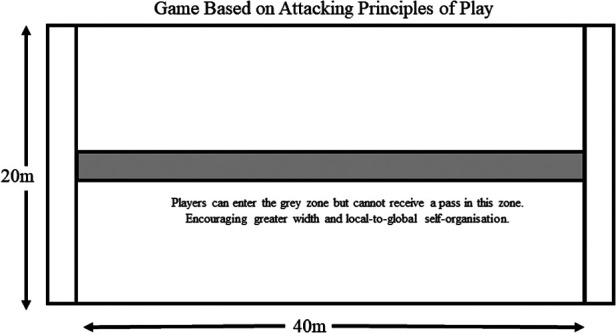
Two teams of 5 vs. 5 play against each other on a standard court, and normal international rules apply. Play is encouraged to practice based on the co-created principles of play [e.g., work in pairs, explore, vary play to play (early pass, change of direction, and tempo); all players stay alive on every play]. Depending on the period and focus of preparation, rules may be included that result in the attacking team maintaining or losing possession if principles are not applied. As indicated in the figure, pitch markings can be added to shape collective team behaviours relevant to the principles of play.

Over time, this approach removed opportunities for dominant players to act as a global influence (because of the fast-paced and multi-dimensional practice conditions). Coaches and support staff encourage stronger local interactions in all players through collectively solving in-game performance challenges to facilitate more even bi-directional synergy formations. This meant that these dynamically changing practice tasks did not look like the game played in competition but required players to quickly adapt their collective behaviours to satisfy interacting individual and task constraints that were co-designed. During this phase of performance preparation, the team transitioned along a continuum of generality (variable practice conditions) (Figure 1) and specificity (representative practice conditions) (Figure 2) of practice conditions. This intentional pedagogical strategy aimed to break the dominant global tendencies and encourage a move towards bi-directional self-organising tendencies.

The combination of general and specific practice tasks facilitated players’ exploration across different performance contexts, where players were observed becoming more responsive to opportunities for interaction, collaboration, and collective team play. The data presented in Table 4 were shared with the players to demonstrate how effective collective play had become in perturbing defensive stability at the point of the tackle and the subsequent defensive stability when the play the ball occurs.

**Table 4:** Defensive stability at PTB and defensive outcome for global-to-local and local-to-global tendencies.

	Tendency					
	Global-to-local	Local-to-global	Global-to-local	Local-to-global	Global-to-local	Local-to-global
	Defensive outcome					
	Great instability		Moderate instability		Stable	
Defensive stability at PTB						
Great instability	0.49% (0)	0.49% (1)	0% (0)	0.49% (1)	0% (0)	0% (0)
Moderate instability	0.49% (1)	0.49% (1)	8.33% (17)	10.78% (22)	3.43% (7)	3.92% (8)
Stable	0% (0)	0.49% (1)	8.33% (17)	9.31% (19)	32.35% (66)	21.08% (43)

Total actions = 204, and data are reported as absolute frequencies and relative frequencies.

### Players’ perceptions of local-to-global self-organising tendencies

Drawing on players’ experiences of performing under localised co-adaptive influences was crucial to developing a better understanding of the application and interplay of bi-directional tendencies. The perceptions of players also highlighted that high levels of physical and mental capacities are required to support effective engagement with this approach, offering important insights for the wider coaching team. It is important to note that performing when guided by a balance of local and global tendencies is termed *GFWS*. This was a principle of play identified by the wider team to encourage better team synergies. Therefore, throughout the next section, local-to-global tendencies are referred to as GFWS.

### Positive experiences

In general, players had positive experiences of GFWS, suggesting that it provided them with more “freedom” (meaning that they perceived that their decision-making and actions were not over-constrained by a rigid game model) to play, helping some players to improve their overall performance. Discussed here by one participant who discussed moving away from a “methodical” way of playing gave players’ more autonomy to play a “free-flowing” style:

Like back when I first started playing people like xxx (player) and xxxxx (player), they wouldn’t have been as good as they are now if they didn’t have the freedom because back in the day like they didn’t have that freedom. Like it was very methodical, if that makes sense, whereas now it’s like throw ball about and see what we can pull off. So yeah, it (GFWS) really opened my eyes up to like having that free-flowing rugby. (P9)

Importantly, participants also reported improved feelings of enjoyment and “confidence” from playing under GFWS. Player 2 also suggested that this way of playing improved the collective attacking ability of the team. He explains:

Yeah, so go forward with support is an easy one to answer for me. I love it. I think it’s brilliant. It’s the way the game used to be played and I think it’s very evident that coming back to that was a good idea. I think our attack look significantly better since we’ve began to correctly implement it and I’m a big fan. I think it’s just a real basic way of playing that brings everyone down to just playing with confidence and enjoying it. (P2)

Participants’ experiences of game-based practice sessions and competition highlighted their perception that defending against GFWS was difficult for opposition teams. This contributed to better *buy in* from the players when experiencing more effective attacking team play. A player explains:

It’s just so difficult to defend and even like the help D (a defensive strategy to implement during periods of instability) it feels a little bit like it’s been designed to defend against go forward with support but even that can’t properly always defend against it, and it doesn’t require like brilliance or anything. It just requires going forward quickly and there’s going to be gaps. (P4)

Players acknowledged that GFWS had superseded more structured elements of attacking phases of play. Importantly, there was a recognition that although GFWS was beneficial to game play, more global influences (structured play) were still important to smooth out periods of instability. Discussed here by player 6:

Like that flamboyancy has like taken over like the structure and the structures good and like certain parts of the game where you know we’re under the cosh, so you play out your sets and you complete your sets then you get back into the game. But yeah, it’s completely opened my eyes to a different side of rugby. (P6)

### “We miss a lot of opportunities to attack”

Although the players report positive experiences with GFWS, they did raise concerns over missing opportunities to attack under this approach. More specifically, when the team was in possession and aiming to advance the ball up field, they felt that the chaotic nature of GFWS meant that opportunities to attack space or exploit numerical advantages (e.g., two attackers vs. one defender) were missed. Highlighted here by player 5:

We miss a lot of opportunities to attack because of the go forward with support, because like you said we have the overlaps on the right but then when we go two people left because it’s like everyone is excited to go and they go left, and we’ve missed the opportunity. (P5)

When playing under GFWS, a principle developed by players and coaching staff was to support the ball carrier with at least one support player, and pass early, before contact is made with a defender. The early pass was aimed at changing the point of attack and creating further opportunities to advance the ball up field. However, as one player identified, this meant that although principles were being applied, sometimes players were too concerned about passing the ball even though it removed the attacking threat. He explains:

We went up together with support, we were doing this, avoid a tackle and then offload it to someone that was right next to us, catch the ball, wouldn’t have much speed so he’s not really a threat. He would keep trying and then pass it again and it would just be constantly passing, passing, passing. (P7)

Players identified that they needed to “quickly recognise” the full range of opportunities for action during game play to enhance collective team attacking play. As one player discussed, adaptability was crucial to becoming better attuned to these opportunities:

As players we need to recognise what’s going wrong and like sort of change, not change the way we play, but quickly recognise what we need to do. (P6)

### A physical and mental challenge

A consequence of playing under GFWS was that physical and mental requirements were heightened. Players reported having to be “switched on all the time” when in possession of the ball because of the necessity to be involved with every play (carrying the ball, supporting the ball carrier, or setting up for the next play). Player 9 discusses this point:

Like you have to be switched on all the time whereas before we’d be like resting on our laurels and go well it’s the third tackle, we’re just over the halfway line, we’ll set one up at four (a specific point on the field) and then we’ll run a set play or whatever. Do you know what I mean? So, it’s a lot tougher like physically and mentally because you’ve got to be switched on all the time, but the results are speaking for themselves aren’t they. (P9)

Player 8 also discusses this point, explaining that attacking play is so “rapid,” highlighting that the selection for interactions between teammates and opposition players and further opportunities for action during practice and competition are a challenge:

Well making decisions a lot quicker now. So, it’s got to be a lot quicker, everybody’s got to know and like you’ve got to see it, see what’s happening, make the decision, let everybody know, like all in the space of like I don’t know like five seconds. Not even that because of how quick the play of the ball is. It’s so like rapid. So, I’d say it’s changed it quite a lot because people like me, xxx (player), xxx (player) who play and make the decisions on the pitch are having to make it a lot quicker. (P8)

### Enhanced team synergy

The emphasis on localised influences on synergy formation during practice provided players with opportunities to collectively explore emergent self-organising tendencies. Players reported that performing under these constraints supported more effective interactions with each other, leading to enhanced team synergies. Discussed here:

Yeah, definitely because it’s not like one person in charge because everyone’s on the same page and there’s no sort of calls to be made. Everyone’s then running sort of how they’d run, and because we’re all doing it so frequently, we’re getting used to each other and knowing when to go (carry the ball or support the ball carrier). (P2)

The comments of the players also suggested that new team synergies (coordination tendencies) emerged under GFWS, where players became more attuned to each other’s future actions. For instance, this player discusses how a reciprocal understanding has been developed between teammates’ intentions through the skill of offloading (passing during contact):

I definitely feel with some players, really changed my interactions as in I know when I’m looking for an offload, I know they’ll be there, or I know they’ll be looking for an offload whereas before I didn’t think of it that way. So, I’ve changed my interactions with them because I think I understand what they’re trying to do on the pitch better. (P10)

It was also highlighted that coordination tendencies were heightened through more awareness of teammate positioning to provide more effective support during attacking opportunities. Exemplified here:

… yeah it definitely keeps you more engaged because you’re not just looking like where should I be, where is my position? It’s where can I be to help them and where can they be to help me, and it just ups that level of communication. (P4)

Player 2 also discussed the development of team synergies, where there was more flexibility to collectively explore performance solutions. The flexible nature of GFWS meant that players did not have to arrive at an exact point on the field in preparation for a set play but rather focused on seeking out opportunities to advance the ball up field:

Rather than it being a clear-cut opportunity all the time (through a structured game plan) it’s more of a sort of decision-making basis (collective interactions under GFWS), and I think because of that people are getting less aggy (annoyed) with each other during games. Which then makes everyone feel better and then they carry on and do it and then they’ll take more risks. (P2)

This example also suggests that stronger team synergies developed the functionality of the team, manifesting through different behaviours (taking more risks), structures (GFWS), and collective intentions (attack space and support the ball carrier).

## Discussion

The overarching purpose of this study was to explore the challenge of knowledge transfer within a high-performance team sports setting for performance preparation. Specifically, the aim was to support players to exploit bi-directional self-organising (inter-individual coordination) tendencies to facilitate emergent attacking behaviours that were contextualised to performance environmental demands. Results from the study highlight several factors that need to be considered when aiming to exchange knowledge in dynamic performance sport settings and provide empirical insights into a bi-directional synergy formation approach in training and performance. First, although challenging, placing transdisciplinary practice at the heart of development and performance environments can facilitate collaboration, thus leading to effective knowledge exchange. Second, implementing new or different knowledge informed by science, or day-to-day actions more generally, is deeply entwined within and influenced by a wider complex system (44). Third, learning systems and knowledge exchange are more effective when key agents within those systems accept that the coach, athlete, and performance context can and should operate through a reciprocal and co-adaptive relationship. In addition, whilst caution is needed when interpreting the results, the qualitative and quantitative data suggest that the bi-directional approach prompts effective learning and performance, where players assumed greater responsibility by actively (re)defining principles of play underlying individual and team performance. This approach can provide players with an increased sense of meaning (they feel part of the process and understand it) in the sense that they recognise the importance of establishing open and flexible principles based on their teammates’ evolving characteristics and the specificities of the context.

These findings highlight that the challenge of knowledge transfer is embedded within the ecology of a complex system, signalling the need for sports scientists and practitioners to reconceptualise the traditional view of knowledge transfer. Situated learning scholars have long argued that knowledge in the form of facts or concepts has little meaning without consideration for the context within which it is intended to be applied (45). For Barab and Roth (46), useful knowledge is an appreciation of and an interaction between facts and concepts and the situations in which they have value. This reconceptualisation of knowledge calls for the rejection “of concepts as self-contained entities and instead conceive of them as tools—tools that can be fully understood only through use” [(46), p. 3]. Therefore, in the sports forum, knowledge transfer should not be viewed as the transmission of “an objective truth” to be imparted on passive recipients but as a contextualised activity reciprocally constructed and shared through continuous functional interactions between athletes, scientists, practitioners, and the performance context. This transactional activity has been termed “correspondence” in the ecological literature (47). Through these situated and contextualised interactions, new performance concepts, ideas, and opportunities can be embedded and explored deep within the ecology of a performance environment. However, when dealing with the challenge of knowledge transfer in context-specific performance environments, institutionalised methods and entrenched beliefs can seem to emphasise an “anti-intellectual agenda,” meaning that positive interactions and, therefore, knowledge creation and transfer can be difficult to achieve [(21), p. 5].

This means that the acceptance, interpretation, and sharing of scientific knowledge and new methods by athletes, coaching practitioners, and sports scientists are socially, culturally, historically, and politically defined. For instance, the cultural, historical, and political backdrop of the team sport of rugby league can shape an individual’s attitude to performance and development, leading to the acceptance and reproduction of practices dominated by top*–*down methods. As Collins (48), Coupland (49), and Rothwell et al. (5) argued, hierarchal systems of control, role specification, and task repetition are attitudes embedded in rugby league’s identity, properties that are synonymous with top*–*down approaches in sports. In complex system theorising, strong context-dependent identities can make the challenge of knowledge transfer difficult. Take the example of England Rugby (50), who have attempted to lower the legal tackle height during competition. Even though changes to the tackle height were based on empirical studies aimed at improving player safety through reduced concussion rates, applying this knowledge into practice has received well-documented backlash from athletes and practitioners (51). This situation highlights that the constitution of scientific knowledge has little meaning when attempts are made to apply it without consideration of context, people, and settings.

As the results highlighted, when individuals within the athlete–coach learning system are adaptive and flexible, more effective and meaningful relations can be fostered. Through these relations, the collective team was more willing to collaborate and share their lived experiences of bi-directional tendencies, opening opportunities for this concept to “*live in* its contextual richness” [(46), p. 3]. This reciprocal approach facilitated a continual coupling of key agents (individuals), content, and context, enabling application, refinement, and opportunities for applied practice to evolve. Crucially, this reciprocity sensitised the wider team to the current performance situation, influencing whether certain components and their function on the overall CAS needed manipulating [e.g. (52)]. In this sense, when an athlete–coach learning system is being challenged to co-adapt through exploring new concepts, a network of *collective affordances* can be generated to evolve individual and team capacities towards a particular task (53, 43). This is evident here with the collective aim of developing more effective attacking synergies (i.e., becoming more proficient at scoring tries). For example, the reciprocal nature of communication, feedback, and interactions highlighted performance issues (e.g., missing opportunities to attack/players recognising what they need to do) that elicited changes to practice. In addition, these meaningful interactions aided the identification of physical and mental development, areas that were acted upon to enhance individual and team performance.

Whilst we recognise that more in-depth analysis of performing under local influences is required, the performance analysis data opened new opportunities for the coaching team and players to collaborate to evolve attacking strategies. This approach is far removed from typical reports of how performance data are used in the coaching process, where athletes are viewed as “objects” and “audience” of the performance analysis process [(54), p. 473]. Athlete insights from Bampouras et al.'s [(54), p. 473/474] study of the in-practice application of performance analysis highlight this trend. An athlete commented:

We were never given the option to say you want to do it or not (performance analysis), how do you think it is going? Is it beneficial towards us or not? We were never given that kind of control.

Manley and Williams (55) argued that employing technology in this way can lead to feelings of anxiety and performance fatigue amongst athletes, in addition to creating barriers to the exchange of knowledge in the athlete–coach–environment learning system [e.g. (56)]. Conversely, using the performance analysis process to inform discussion points, practice designs, and the evolution of principles of play can foster learning environments where exchanging knowledge between athletes and coaches is the norm [for an excellent applied example, see (57)]. This study used data to support coaches’ and players’ understanding of the value of moving along a continuum of global and local self-organising tendencies to create instabilities within the defensive system.

Crucially, the performance analysis process and subsequent data set strengthened interactions between the players, coaches, and contents and focused player intentions towards specifying information sources and periods of play that presented opportunities to attack. The performance analysis data could suggest that flexible principles of play provide players with opportunities to adapt and refine novel performance solutions, evident through the destabilisation of defensive team structures [e.g. (30)]. Therefore, principles of play pertaining to a game model could be used to potentiate the evolving characteristics of the players through continuous skill-based engagement with a rich landscape of affordances. It should be noted that principles of play are the beginning of pattern or synergy formation and through a flexible approach should encourage players to recreate a particular principle as they develop an increasingly functional fit with the performance environment (58). Individual and team coordination patterns are not pre-determined, re-wired, or mechanised. Rather, players need to continually search for and utilise specifying information to accomplish principles of play. Through practice, players must be given time and space to explore and understand principles and operationalise them without constant monitoring and feedback or through the prescription of mechanised and repetitive performance solutions.

A fundamental point to be outlined here is that a game model allows the wider coaching team and athletes to structure and organise their practices around a framework of intentions according to the development of specific principles of play that form a team’s identity. This intentionality framework could play to collective strengths and minimise the effects of weaknesses. Therefore, the game model must always be available to guide the players’ attention and collective intentions (42) during the performance preparation process. This could be predicated on coaches organising and structuring practice designs to develop intended performance outcomes, coherent with the fulfilment of principles of play for attacking and defending phases. In this sense, used as a guiding framework, the game model or tactical principles of play are never the problem. Rather, the problem lies in how coaching practitioners and sports scientists conceive and use them in a highly prescriptive way.

## Limitations and future directions

It is important to acknowledge that a small body of research indicates that some practitioners are already beginning to successfully transfer knowledge into applied settings (59, 57). These examples have emerged in both individual (e.g., paddle sports) and team sports (rugby union) contexts. Future research is needed to continue to establish how practitioners could achieve knowledge transfer in elite sport performance contexts. Studies of this nature would supplement the findings from the qualitative element of this paper to provide important insights for both academics and practitioners, as it seems that the goal of knowledge transfer remains problematic. More specifically, research could distinguish between “what” decisions are made and “how” decisions are implemented regarding the application of empirical knowledge (60). The “what” knowledge represents the empirical knowledge that will help underpin the design and implementation of alternative performance preparation practices. These studies can also outline “how” these practices are delivered by practitioners and experienced by athletes. Employing qualitative research methods, such as observations, interviews, and case studies, can verify key agents’ lived experiences of the athlete–coach–environment learning system. To clarify, we are not suggesting the formulation of another methodology but rather the identification of principles that can promote the discovery of new synergies, not only in the player and team performance sense but also amongst academics, practitioners, and athletes, to facilitate the better application of sports science research.

Whilst the performance analysis data from this study focused more on opening new opportunities for co-adaptation between the coaching team and players, further insights could be crucial to understanding the value of moving between bi-directional tendencies. This case study examined a match sample from four games. Future research could address the feasibility of transitioning between bi-directional tendencies from various team sports using short-term (<6 weeks) intervention designs. Examining multiple teams would enable researchers to conduct inferential statistical analysis to explore the findings of a population beyond a narrow sample. Moreover, this recommendation for inferential statistical analysis is pertinent for knowledge transfer, given that the differences between “global-to-local” and “local-to-global” tendencies in this study were minimal. It is important to acknowledge that the coaching period of each condition in this study was unbalanced, with players receiving more time in the “global-to-local” condition coaching because of previous coaching and cultural practices of the national team. This tendency to direct the majority of practice towards more “global-to-local tendencies” in forming synergetic relations between players in training could further prove an over-valuation of “global-to-local” tendencies in team sports.

## Data Availability

The raw data supporting the conclusions of this article will be made available by the authors, without undue reservation.
